# Reducing Sun Exposure for Prevention of Skin Cancers: Factorial Invariance and Reliability of the Self-Efficacy Scale for Sun Protection

**DOI:** 10.1155/2015/862732

**Published:** 2015-09-17

**Authors:** Steven F. Babbin, Hui-Qing Yin, Joseph S. Rossi, Colleen A. Redding, Andrea L. Paiva, Wayne F. Velicer

**Affiliations:** ^1^Department of Psychiatry, Geisel School of Medicine at Dartmouth, Lebanon, NH 03766, USA; ^2^Cancer Prevention Research Center and Department of Psychology, University of Rhode Island, Kingston, RI 02881, USA

## Abstract

The Self-Efficacy Scale for Sun Protection consists of two correlated factors with three items each for Sunscreen Use and Avoidance. This study evaluated two crucial psychometric assumptions, factorial invariance and scale reliability, with a sample of adults (*N* = 1356) participating in a computer-tailored, population-based intervention study. A measure has factorial invariance when the model is the same across subgroups. Three levels of invariance were tested, from least to most restrictive: (1) Configural Invariance (nonzero factor loadings unconstrained); (2) Pattern Identity Invariance (equal factor loadings); and (3) Strong Factorial Invariance (equal factor loadings and measurement errors). Strong Factorial Invariance was a good fit for the model across seven grouping variables: age, education, ethnicity, gender, race, skin tone, and Stage of Change for Sun Protection. Internal consistency coefficient Alpha and factor rho scale reliability, respectively, were .84 and .86 for Sunscreen Use, .68 and .70 for Avoidance, and .78 and .78 for the global (total) scale. The psychometric evidence demonstrates strong empirical support that the scale is consistent, has internal validity, and can be used to assess population-based adult samples.

## 1. Introduction

Skin cancer is a widespread, growing, and costly public health problem. Nonmelanoma skin cancers, which include squamous cell carcinoma and basal cell carcinoma, are the most common malignancies in the United States, with approximately 3.5 million new cases diagnosed each year [1]. Melanoma, although less common, is the deadliest form of skin cancer. Nearly 60,000 people are diagnosed and about 8,600 people die from melanoma each year [2]. Overall, skin cancer is the most common form of cancer in the United States, and incidence is increasing [1]. It results in significant potential years of life lost and billions of dollars in costs, including both medical costs and lost productivity costs [3, 4].

The burden of skin cancer can be reduced through prevention efforts. Exposure to ultraviolet radiation via the sun is the most important and avoidable cause of skin cancers. Unfortunately, the majority of adults in the United States do not protect themselves from the sun, and prevalence of at least one sunburn per year is over 50% [5, 6]. Sun protection behaviors, such as avoiding the sun, wearing protective clothing, and wearing sunscreen, can be emphasized in interventions designed to increase sun protection.

The Self-Efficacy Scale for Sun Protection was developed using the framework of the Transtheoretical Model of Behavior Change (TTM). The TTM is an integrative framework that consists of multiple dimensions that assess readiness to change [7, 8]. The core constructs of the TTM include stages of change, processes of change, decisional balance, and self-efficacy. Tailored, computerized interventions based on the TTM have been empirically validated and effective for a wide variety of behaviors [9–11], including sun protection behaviors [12–16].

The self-efficacy component of the TTM assesses an individual's perceived ability to perform healthy behaviors in difficult situations [17]. This component is based on self-efficacy as conceptualized by Bandura [18] and research on relapse prevention. The Self-Efficacy Scale for Sun Protection was designed to assess an individual's confidence to protect oneself from sun exposure [6, 19, 20]. Variations of this measure have been utilized in multiple population-based interventions that have demonstrated efficacy and effectiveness (e.g., [12–16]).

In a tailored intervention, different response patterns to the self-efficacy scale result in different, individualized feedback for participants. To confirm that the Self-Efficacy Scale for Sun Protection is useful and meaningful for intervention purposes across a wide range of potential target populations, the psychometric assumptions of factorial invariance and scale reliability were evaluated in the present study with a large, representative sample of adults (*N* = 1356) from across the United States involved in a tailored intervention study for exercise and sun protection. The psychometric assumptions of measurement invariance and scale reliability are crucial to the overall construct validity of the measure. A measurement model is called* factorially invariant* when the model is the same for different subgroups of a population. Three levels of factorial invariance, from the least restrictive to the most restrictive, were assessed. These levels have been used to test invariance of other TTM constructs [21–24]. Factorial invariance for the Decisional Balance for Sun Protection Inventory, a measure that was used in the same intervention study that provided data for the present study, was also tested with these three levels [25].

Each level adds more constraints to the model. The weakest level is* Configural Invariance*, which states that subgroups have zero loadings on the same constructs and unconstrained nonzero factor loadings [26]. Second is* Pattern Identity Invariance*, which requires the factor loadings to be equal across subgroups. Third,* Strong Factorial Invariance* requires factor loadings and error terms to be equivalent across subgroups. If a scale is factorially invariant across groups, the psychometric properties of the measure can be assumed equal across groups (e.g., factor correlations and internal consistency). Thus, comparisons between groups on the measure of interest can confidently be attributed to true differences in the construct and not simply to variance on the measure.

The present study was a secondary data analysis of a baseline sample of adults involved in a tailored intervention study. Factorial invariance was examined across age, education, ethnicity, gender, race, skin tone, and Stage of Change for Sun Protection. Internal consistency reliability of the scales was assessed with Cronbach's Coefficient Alpha. Scale reliability, based on results from confirmatory factor analysis (CFA), was assessed with the factor rho coefficient.

## 2. Methods

### 2.1. Participants

Baseline data from 1356 adults were used for this study. These participants were recruited from across the United States in 2010–2012 for a randomized, population-based, TTM-tailored intervention study [27]. The study evaluated a multimedia, computer-based intervention to increase exercise and sun protection. Recruitment was proactive, and participants were identified with screening calls. To be eligible for the study, participants had to be in preaction stages (precontemplation, contemplation, or preparation; [7, 8]) for both exercise and sun protection; eligible participants were not currently exercising and not currently protecting themselves from the sun. Additional inclusion criteria included age 18–75, willingness to provide basic demographic information, ability to participate in physical activity, and internet access. Participants that had a recent history of medical conditions (e.g., heart attack within the past six months, currently receiving chemotherapy or radiation, and pregnant women) or who had been advised by their doctor or health care provider to avoid exercise were excluded. All intervention materials and assessments were administered over the internet. Consent and other human subject protocols were approved by the University of Rhode Island Institutional Review Board, and research was conducted according to APA ethical guidelines. Demographic variables were utilized to create subgroups for invariance testing (see Table 1). Overall, the sample was 83% white and 63% female.

### 2.2. Self-Efficacy Scale for Sun Protection

**Figure 1:**
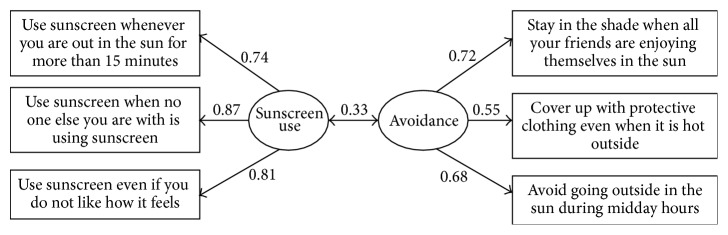
Model with standardized parameter estimates for the total sample (*N* = 1356).

The Self-Efficacy Scale for Sun Protection was designed to assess confidence in sun protection behaviors in adolescents and adults [6, 19, 20, 28] and was developed using the sequential method of scale development [29–31]. The scale can be modeled as a two-factor correlated model with six items: three items for Sunscreen Use and three items for Sun Avoidance (see Figure 1). For each item, participants rated how confident they were in their ability to protect themselves from sun exposure on a 5-point Likert scale, from 1, “not at all confident,” to 5 “extremely confident.” The scale can also be scored as a global measure of self-efficacy for sun protection by averaging (or summing) all six items into a single total score. In the computer-based assessments, participants could not skip scale items. Thus, there were no missing data from item nonresponse in this baseline sample.

### 2.3. Factorial Invariance

Three levels of invariance were tested in sequential order, with each level requiring more constraints: (1) Configural Invariance (unconstrained nonzero factor loadings); (2) Pattern Identity Invariance (equal factor loadings); and (3) Strong Factorial Invariance (equal factor loadings and measurement errors). Each invariance procedure was evaluated across specific subgroups.

Baseline variables were used to create subgroups (see Table 1). In general, when continuous variables were divided into categories (e.g., age and education), the goal was to avoid subgroup sizes of <100 to avoid convergence issues [32]. For other variables, subgroups that were too small for analysis were eliminated. Only complete cases were used for these subgroups. Thus, these subgroups vary in total sample size due to differences in missing data across subgroup variables. The sample was divided into five subgroups for age, three subgroups for education, two subgroups for ethnicity, two subgroups for gender, two subgroups for race, three subgroups for skin tone (untanned skin color, a proxy indicator of sun sensitivity [19]), and three subgroups for Stage of Change for Sun Protection (see Table 1). Despite the very small sample size of participants identified as Hispanic, the sample size was adequate for analysis. Demographic questions included more racial identities (American Indian or Alaskan Native, Asian, Native Hawaiian or other Pacific Islanders, and others), but no other subgroups had adequate sample sizes for analysis. Sample size for participants who identified skin tone as dark brown was not adequate for invariance testing (*n* = 44). Stage of Change for Sun Protection is a TTM construct that represents readiness to change sun protection behavior [6, 19]. The precontemplation stage included participants who were not consistently protecting themselves from the sun and were not intending to start within the next 12 months. The contemplation stage included participants who were not consistently protecting themselves but were seriously thinking about starting within the next 12 months. Preparation stage individuals were not currently protecting themselves but were planning to start within the next 30 days.

To test for factorial invariance, structural equation modeling (SEM) was employed using EQS 6.1 software [33]. Model fit was evaluated using the comparative fit index (CFI) and the root mean square error of approximation (RMSEA). For CFI, values closer to 1.0 indicate good fit and, for RMSEA, values closer to zero indicate good fit [34, 35]. The difference in CFI between a higher level model and a lower level of invariance (ΔCFI) was also calculated (e.g., Pattern Identity CFI-Configural CFI). A difference of .01 or smaller indicates that the null hypothesis of invariance should not be rejected and that the model demonstrates invariance [36]. The fit indices and invariance modeling procedures used in the present study are the same as included in recent psychometric assessments of self-efficacy measures for other behaviors [22, 23].

### 2.4. Scale Reliabilities

The internal consistency reliability of each subscale (Sunscreen Use and Sun Avoidance) and the total scale was assessed with Cronbach's Coefficient Alpha [37]. Confidence intervals for coefficient Alpha were also calculated [38]. The factor rho coefficient [34, 39–41] was calculated for each subscale and the total scale to assess scale reliability based on CFA results. Unstandardized model estimates were used to calculate rho. For both estimates of reliability, there are no strict cutoffs for acceptability [42], but values around .70 indicate adequate internal consistency, and values around .90 indicate excellent internal consistency [31, 34].

## 3. Results

### 3.1. Factorial Invariance

A total of 21 models were run, with three models (Configural Invariance, Pattern Identity Invariance, and Strong Factorial Invariance) for each of the seven subgroup variables. No constraints were dropped in any of the models to achieve a better fit. All sample sizes and results are summarized in Table 2.

#### 3.1.1. Age

Sample size for invariance testing was adequate for the five subgroups: 18 to 29 years old, 30 to 39 years old, 40 to 49 years old, 50 to 59 years old, and 60 years old and over. The highest level of invariance, Strong Factorial Invariance, held across the subgroups with a good fit (CFI = .966; RMSEA = .066).

#### 3.1.2. Education Level

Sample size was adequate for all three subgroups: 12 years of education or less, 13 to 15 years of education, and 16 years of education or more. Strong Factorial Invariance held across the subgroups with a good fit (CFI = .971; RMSEA = .063).

#### 3.1.3. Ethnicity

Sample size was marginal for the Hispanic group, especially as compared to the non-Hispanic group, but there were no difficulties in fitting the models; thus, the sample size for Hispanics was considered acceptable. Strong Factorial Invariance fit well across the subgroups (CFI = .977; RMSEA = .060).

#### 3.1.4. Gender

Sample size was adequate for both subgroups: female and male. Strong Factorial Invariance held across the subgroups (CFI = .968; RMSEA = .070).

#### 3.1.5. Race

As with ethnicity, sample size was marginal but acceptable for the black/African American group and was large for the white subgroup. Strong Factorial Invariance fit well across the subgroups (CFI = .973; RMSEA = .064).

#### 3.1.6. Skin Tone

Sample size was adequate for three subgroups: fair white, medium white, and dark white/light brown. Strong Factorial Invariance held across the three subgroups with a good fit (CFI = .935; RMSEA = .091).

#### 3.1.7. Stage of Change for Sun Protection

Sample size was adequate for the three subgroups: precontemplation, contemplation, and preparation. Strong Factorial Invariance held across the subgroups (CFI = .953; RMSEA = .071).

### 3.2. Scale Reliabilities

Since Strong Factorial Invariance held for all of the subgroups, a CFA was performed on the total sample, and the final correlated model structure and parameter estimates are reported only for the total sample (see Figure 1). The total sample coefficient Alpha [95% confidence interval] was .84 [.82, .85] for Sunscreen Use, which indicates very good internal consistency reliability, and .68 [.65, .71] for Avoidance, which indicates adequate internal consistency reliability. Coefficient Alpha for the total six-item scale was good, *α* = .78 [.76, .80]. The CFA-based factor rho coefficient was .86 for Sunscreen Use, which indicates very good scale reliability, .70 for Avoidance, which indicates adequate scale reliability, and .78 for the total six-item scale, which indicates good scale reliability. Considering the short length of the scale, the Alpha values, and the rho values, the Self-Efficacy Scale for Sun Protection demonstrates evidence of reliability.

## 4. Discussion

Assessments of factorial invariance and internal consistency suggest that the Self-Efficacy Scale for Sun Protection is a reliable and valid instrument and can be used across a full range of adult participants varying by age, educational level, skin tone, gender, ethnicity, race, and stage attributes. The scale demonstrates a high level of factorial invariance across subgroups. The highest level tested, Strong Factorial Invariance, required that factor loadings and error terms were equal across the subgroups. This demonstrated a good fit across age, education level, ethnicity, gender, race, skin tone, and Stage of Change for Sun Protection. Internal consistency reliability, as assessed by coefficient Alpha, was very good for the Sunscreen Use subscale, adequate for the Avoidance subscale, and good for the total six-item scale. Scale reliability, as assessed by the factor rho coefficient, was also very good for the Sunscreen Use subscale, adequate for the Avoidance subscale, and good for the total six-item scale. Overall, these results suggest that there is a consistent relationship between the two subscales (Sunscreen Use and Avoidance), as well as the six items that measure these factors.

The consistently good fit for Strong Factorial Invariance across seven subgrouping variables is very strong evidence that the scale is factorially invariant. The degree of fit does vary across the subgroups, however. For age, education level, ethnicity, gender, and race, CFI, ΔCFI, and RMSEA all indicated good fit for Strong Factorial Invariance. For skin tone and Stage of Change for Sun Protection, CFI and RMSEA suggested good fit, but the ΔCFI value was slightly more negative than −.01. This suggests that there may be some small differences in the factor model of the scale across levels of skin tone and Stage of Change for Sun Protection. But since most of the indices suggested good fit, Strong Factorial Invariance should not be rejected.

The high level of factorial invariance refers to the consistency in the factor structure. Such consistency in the measurement model is vital to valid research and intervention efforts. Since a population-based sample cannot be homogenous in every way, every sample will demonstrate some variation that is not related to the research question. Some subgroups may respond to the measurement instrument differently. Results from a factorially invariant model should not be biased by such differences. Strong Factorial Invariance suggests that the validity of the measurement should be the same regardless of the sample. However, since the focus is on the factor structure, the means were not assessed for equivalence across subgroups. Mean differences, in some cases, could be expected to be different. For example, one would anticipate that participants in the precontemplation stage for sun protection (not intending to start protecting themselves from the sun within the next 12 months) would have lower mean scores for confidence in Sunscreen Use and Avoidance than participants in the contemplation stage for sun protection (thinking about starting within the next 12 months). Testing such mean differences could be the focus of a future study.

The Self-Efficacy Scale for Sun Protection was developed to be consistent with self-efficacy theory [18]. The six items of the scale represent six different situations that represent obstacles and challenges to an individual's perceived ability to protect himself or herself from the sun. In general, self-efficacy scales developed for TTM studies involve a variety of situations related to one target behavior, such as smoking [17, 23] or alcohol use [22]. The Self-Efficacy Scale for Sun Protection, in contrast, involves multiple sun protection behaviors (wearing sunscreen, avoiding the sun, and wearing protective clothing). In the construction of the scale, including multiple behaviors was necessary due to the complex nature of sun protection; to be protected from the sun, an individual needs to perform multiple sun protection behaviors [6, 19, 20, 28]. Thus, this scale is more behaviorally defined and more operationalized in terms of behaviors than some other scales. Evidence from factorial invariance suggests that this larger emphasis on behavior does not impact the generalizability of the factor structure. Strong Factorial Invariance held across all subgroups, and this suggests that participants in different subgroups did not respond differently to items involving different behaviors. Regardless, the behaviorally defined nature of the scale may have some impacts on the measurement of self-efficacy that are beyond the scope of the present study but could be explored in future measurement studies. For example, some items related to specific behaviors may be more predictive of treatment outcome.

While reliability for the Confidence in Sunscreen Use subscale was good, reliability for the Avoidance subscale could be improved. Coefficient Alpha and coefficient rho were close to .70, which suggests that reliability was only adequate. The simplest solution to improving reliability would be to add items to the Avoidance subscale, as longer scales demonstrate greater reliability. However, the Self-Efficacy Scale for Sun Protection was intended to be brief, and an increase in reliability via more items would increase the time required to answer the scale and decrease the parsimony of the model. Past psychometric assessments of short TTM scales [21–25] have reported coefficient Alpha values ranging from .51 to .90, which suggests that an Alpha around .70 is consistent with similar measures. In addition to the two subscales, many applied researchers are likely to use the global (total) scale score as well, as an assessment of overall confidence in the ability to perform protective sun behaviors. The two measures of reliability, coefficient Alpha and coefficient rho, were good (nearly .80) for the total scale score. Both the total score and the subscale scores can be useful as intermediate level indicators of intervention effectiveness, prior to final intervention outcome assessments of behavior change [7, 8, 17].

The psychometric assessment of this scale was limited by some subgroup sample sizes. Most importantly, three subgroup variables could be improved with a larger, more diverse sample: ethnicity, race, and skin tone. The number of participants that identified their ethnicity as Hispanic was very small, and, therefore, the results of the invariance tests for ethnicity should be interpreted with caution. The number of participants identified as black or African American was also small. In addition to improving the sample size of this subgroup, a larger sample could cover a more comprehensive number of racial identities. Insufficient sample size also prevented participants who identified “dark brown” skin tones from being included in separate invariance analyses. A larger sample would improve the respective sample sizes for ethnicity, race, and skin tone and thus provide stronger evidence for validity.

The present study focused on invariance, internal consistency reliability, and scale reliability. These properties do not encompass all aspects of validity and reliability, and future investigations could test other psychometrics using assessment points beyond baseline. Test-retest reliability, or the stability of the measure over time, needs to be assessed. As the intervention was designed to promote sun protection, the factor means should change, but the overall factor structure should remain stable. Convergent validity could be assessed by comparing self-efficacy scores to self-reported sun protection behaviors at each assessment point. Predictive validity could be assessed by testing baseline self-efficacy scores as predictors of sun protection behaviors at future assessment points. Such efforts would further strengthen the evidence supporting the validity and reliability of this measure.

## 5. Conclusions

The Self-Efficacy Scale for Sun Protection demonstrates consistency and reliability. Ultimately, assessment of these psychometric properties provides strong empirical evidence for construct validity. This scale measures self-efficacy for sun protection as intended. It is short, psychometrically sound, and appropriate for research, providing a solid empirical foundation for the development of interventions to reduce the burden of skin cancer.

## Figures and Tables

**Table 1:** Sample sizes for subgroups included in invariance testing.

Variable Subgroup	*n*
Age (years)	
18–29	188
30–39	202
40–49	353
50–59	360
≥60	253
*Total*	*1356*
Education level (years)	
≤12	313
13–15	468
≥16	575
*Total*	*1356*
Ethnicity	
Hispanic	54
Non-Hispanic	1302
*Total*	*1356*
Gender	
Female	856
Male	500
*Total*	*1356*
Race	
White	1123
Black/African American	89
*Total*	*1212*
Skin tone (untanned skin color)	
Fair white	296
Medium white	598
Dark white (e.g., olive) and light brown	406
*Total*	*1300*
Stage of Change for Sun Protection	
Precontemplation	836
Contemplation	152
Preparation	367
*Total*	*1355*

**Table 2:** Goodness-of-fit statistics for three invariance models.

	Model	*χ* ^2^	df	CFI	ΔCFI	RMSEA	[90% CI]
Age	Configural Invariance	112.424	40	.973	—	.082	[.064, .100]
	Pattern Identity Invariance	142.684	56	.968	−.005	.076	[.060, .091]
	Strong Factorial Invariance	173.593	80	.966	−.002	.066	[.052, .079]

Education	Configural Invariance	86.280	24	.977	—	.076	[.059, .093]
	Pattern Identity Invariance	99.994	32	.975	−.002	.069	[.054, .084]
	Strong Factorial Invariance	122.958	44	.971	−.004	.063	[.050, .076]

Ethnicity	Configural Invariance	81.672	16	.976	—	.078	[.061, .095]
	Pattern Identity Invariance	83.246	20	.976	.000	.068	[.053, .084]
	Strong Factorial Invariance	88.732	26	.977	.001	.060	[.046, .073]

Gender	Configural Invariance	91.839	16	.972	—	.084	[.067, .100]
	Pattern Identity Invariance	97.756	20	.971	−.001	.076	[.061, .091]
	Strong Factorial Invariance	112.064	26	.968	−.003	.070	[.057, .083]

Race	Configural Invariance	67.817	16	.978	—	.073	[.056, .091]
	Pattern Identity Invariance	69.539	20	.979	.001	.064	[.048, .080]
	Strong Factorial Invariance	89.886	26	.973	−.006	.064	[.049, .078]

Skin tone	Configural Invariance	124.397	24	.958	—	.098	[.081, .115]
	Pattern Identity Invariance	155.343	32	.948	−.010	.094	[.080, .109]
	Strong Factorial Invariance	201.558	44	.935	−.013	.091	[.078, .104]

Stage of Change	Configural Invariance	79.228	24	.974	—	.071	[.054, .089]
	Pattern Identity Invariance	88.925	32	.973	−.001	.063	[.047, .078]
	Strong Factorial Invariance	145.220	44	.953	−.020	.071	[.059, .084]
